# Integrated pharmacology and experimental validation reveals potential multiple mechanisms of the neutrophil elastase inhibitor sivelestat in attenuating myocarditis

**DOI:** 10.3389/fphar.2026.1695352

**Published:** 2026-04-30

**Authors:** Ruguo Ren, Ning Wang, Chen Chen, Miao Yang, Bin Li, Yuxuan Yang, Hang Zhang, Bo Yang, Yu Wu, Wei Gao, Yuanyuan Hou

**Affiliations:** 1 Department of Anesthesiology, The First Affiliated Hospital of Xi’an Jiaotong University, Xi’an, Shaanxi, China; 2 Department of Cardiothoracic and Vascular Surgery and Critical Care Medicine, Xi’an No.1 Hospital and The First Affiliated Hospital of Northwest University, Xi’an, Shaanxi, China; 3 Department of Anesthesiology, The First Affiliated Hospital of Anhui Medical University and Anhui Public Health Clinical Center, Hefei, Anhui, China; 4 Cornerstone of Health Technology (Shaanxi) Co., Ltd., Xianyang, Shaanxi, China; 5 Department of Anesthesiology, Xi’an Chest Hospital and Northwest University Affiliated Chest Hospital, Xi’an, Shaanxi, China

**Keywords:** Balb/c mice, immune myocarditis, molecular docking, network pharmacology, neutrophil extracellular traps (NETs), PI3K-Akt pathway, sivelestat

## Abstract

**Background:**

Myocarditis is an inflammatory cardiomyopathy characterized by high level of inflammatory cell infiltration and progressive cardiac dysfunction. The underlying pathogenesis involves direct pathogen damage and inflammation-mediated tissue destruction. Previous studies showed the therapeutic potential of Sivelestat (the neutrophil elastase inhibitor) in mice models, but the underlying mechanisms remain elusive.

**Objective:**

To elucidate the cardioprotective mechanisms of Sivelestat, a neutrophil elastase inhibitor, in experimental autoimmune myocarditis (EAM).

**Methods:**

Network pharmacology identified shared targets between Sivelestat and myocarditis. Molecular docking validated binding affinities. EAM was induced in male BALB/c mice (n = 6/group) using α-myosin heavy chain peptide. Interventions included Sivelestat sodium (50/100/200 mg/kg/day i. p., 14 days). Cardiac function (echocardiography), inflammation (serum IL-1β/IL-6/TNF-α/cTn; histopathology; immunohistochemistry for myocardial IL-6, IL-1β, and TNF-α), NETosis (Cit-H3/NE immunofluorescence/Western blot/SEM), apoptosis (TUNEL), and PI3K-Akt signaling (Western blot) were assessed.

**Results:**

Computational analysis identified 41 potential targets, highlighting the PI3K-Akt and IL-17 signaling pathways as top candidates. High-affinity binding was confirmed for key targets (e.g., *PTGS2*: −9.0 kcal/mol). *In vivo* administration of Sivelestat (200 mg/kg) significantly improved left ventricular function and fractional shortening, and reduced serum levels of inflammatory cytokines (IL-1β, IL-6, TNF-α) and cardiac troponin (cTn). Immunohistochemical analysis confirmed that Sivelestat significantly reduced the myocardial expression of IL-6, IL-1β, and TNF-α consistent with the serum findings and further demonstrating its local anti-inflammatory effects. Moreover, qPCR validation demonstrated that Sivelestat significantly downregulated the mRNA expression of the network pharmacology-predicted targets *TNF*, *MMP9*, *PTGS2*, and *IL-17* in myocardial tissue, providing transcriptional evidence supporting the multi-target mechanism. Additionally, Sivelestat decreased cardiomyocyte apoptosis through activation of the PI3K-Akt pathway, as demonstrated by increased p-Akt and Bcl-2, and decreased cleaved caspase-3.

**Conclusion:**

Sivelestat mitigated myocarditis through multiple mechanisms, including immunomodulatory effects (NETosis inhibition and *PTGS2* inhibition mediated IL-17A Pathway modulation) and PI3K-Akt-mediated anti-apoptosis. Due to the multi-target action and established clinical safety profile, Sivelestat had the potential for rapid clinical translation.

## 1 Introduction

Myocarditis is a complex inflammatory disease of the myocardium, often resulting from various etiological factors including viral infections, autoimmune diseases, or drugs. It may lead to heart failure or sudden cardiac death (Basso, 2022; Brociek et al., 2023). Current therapies, including corticosteroids, immunosuppressants, and antiviral treatments. However, these approaches are not universally effective and can be associated with limitations, including incomplete efficacy in a subset of patients and potential side effects from broad immunosuppression (Basso, 2022; Brociek et al., 2023; Lasica et al., 2023; Ammirati and Moslehi, 2023; Yakhshimurodov et al., 2024). The primary cause of myocarditis is viral infection, which progresses through three stages: direct viral damage, immune damage, and the Dilated Cardiomyopathy (DCM) phase. The most detrimental aspect for patients with viral myocarditis is immune myocardial injury from autoimmune myocarditis (AM). AM occurs due to immune dysfunction, leading to antibody-antigen complexes that infiltrate the myocardium, causing severe inflammation, cell damage, necrosis, edema, and monocyte infiltration, further triggering an autoimmune response (Tschope et al., 2021; Barcena et al., 2021). Persistent immune damage to the heart muscle significantly contributes to the development of viral myocarditis into DCM. The exact cause of AM remains unclear, and effective treatments are lacking. Thus, researching therapies for AM is crucial for improving myocarditis patient outcomes. Sivelestat is a specific neutrophil elastase (NE) inhibitor approved for the treatment of acute lung injury (ALI) associated with systemic inflammatory response syndrome (Ding et al., 2023). Its clinical use is generally well-tolerated, with reported adverse effects including transient liver enzyme elevations and rash, though these are relatively infrequent. However, its role in cardiac inflammation and myocarditis remains insufficiently investigated. The novelty of this study lies in the systematic integration of computational and experimental approaches to elucidate the multi-target mechanisms of Sivelestat in immune myocarditis, moving beyond its known role as a NETosis inhibitor.

In this study, we first investigated Sivelestat’s potential targets and signaling pathways in myocarditis treatment. As a NE inhibitor which inhibits Neutrophil Extracellular Traps (NETs) formation by blocking NE proteolytic function and nuclear localization (Lim et al., 2024; Tokuyama et al., 2021; Huang et al., 2022; Wang H. et al., 2024). Emerging evidence suggests that NETs involve in cardiac inflammation and myocardial damage in AM (Carai et al., 2023; Weckbach et al., 2019; Li et al., 2022; Ichimura et al., 2024; Du et al., 2020; Zhang et al., 2025). Therefore, inhibition of NETosis by Sivelestat may ameliorated cardiac fibrosis and function in mice with experimental autoimmune myocarditis (EAM). Moreover, network pharmacology and molecular docking further found more targets and validated through *in vivo* experiments, including PTGS2 inhibition that involves IL-17A Pathway modulation and apoptosis inhibition by PI3K-Akt pathway activation. The findings of this study provide a robust scientific basis for future clinical management of myocarditis. The primary aim of this study was to comprehensively elucidate the cardioprotective mechanisms of Sivelestat in EAM using an integrated strategy combining network pharmacology, molecular docking, and experimental validation. We planned to achieve this by first predicting Sivelestat’s potential targets and pathways computationally, and then rigorously validating these predictions in a well-established EAM mouse model through assessments of cardiac function, inflammation, NETosis, apoptosis, and key signaling pathways.

## 2 Materials and methods

### 2.1 Targets prediction of sivelestat and myocarditis

The structural information of Sivelestat was obtained from the PubChem database (https://pubchem.ncbi.nlm.nih.gov/). The canonical Simplified Molecular Input Line Entry System (SMILES) notation for Sivelestat was subsequently imported into the SwissTargetPrediction (http://swisstargetprediction.ch/) and SEA (https://sea.bkslab.org/) databases to predict its potential targets. In order to identify candidate genes with high confidence, we adopted strict screening criteria. From SwissTargetPrediction, we screened the genes with probability score >0, which ensured that all the genes included in the analysis were statistically significant (*P* < 0.05). From SEA databases, we screened genes with maxTC value >0.3. Finally, we define the genes that meet both criteria as overlapping candidate genes for further analysis. The keyword “myocarditis” was used to search the GeneCards (https://www.genecards.org/) and OMIM (https://omim.org/) databases to identify relevant disease targets. Similarly, the keyword “neutrophil extracellular traps” was employed to locate pertinent NETs network targets within the GeneCards and OMIM databases. The disease targets obtained from these databases were consolidated, and duplicate entries were removed to establish a final set of disease targets. The identified drug targets, NETs targets, and disease targets were then cross-referenced. A Venn diagram was generated using Venny (https://bioinfogp.cnb.csic.es/tools/venny/) to identify intersecting genes. Finally, a drug-target network was constructed using Cytoscape 3.10.3.

### 2.2 Construction of protein–protein interaction network

To further investigate protein-protein interactions (PPIs) in Sivelestat-treated myocarditis, drug-intersection genes were uploaded to the STRING interaction database (https://stringdb.org/) for PPI network construction. The species was specified as *Homo sapiens*, with other parameters maintained at their default settings. The resulting data were saved in tab-separated value (TSV) format and subsequently imported into Cytoscape 3.10.3. Core targets were identified using undirected network metrics (betweenness, closeness, and degree) through CentiScaPe 2.2 analysis. Targets scoring above the mean value for each metric were selected and intersected to determine Sivelestat’s essential therapeutic targets for myocarditis. Betweenness centrality (measuring how often a node lies on the shortest paths) and closeness centrality (measuring the average path length to other nodes) indicate a node’s network connectivity, with higher values representing greater topological importance. Node size and color intensity reflected degree values, where larger nodes corresponded to higher degree values. These key targets were used to construct the final PPI network diagram.

### 2.3 Enrichment analysis of gene ontology (GO) function and kyoto encyclopedia of genes and genomes (KEGG) pathway

The identified intersection genes were uploaded to the DAVID database (https://david.ncifcrf.gov/summary.jsp) for further analysis. GO analysis and KEGG pathway enrichment analysis were conducted, employing a significance threshold of *P* < 0.05 as the criterion for screening. Utilizing the GO annotation system provided by DAVID 6.8, we examined the functional roles of Sivelestat target proteins in the treatment of myocarditis, with a focus on biological processes (BP), cellular components (CC), and molecular functions (MF). Subsequently, a KEGG pathway analysis was performed to identify potential therapeutic targets of Sivelestat in myocarditis. The top-ranked pathways were visualized using the bioinformatics platform (https://www.bioinformatics.com.cn/). A literature review was then conducted for the top 20 KEGG pathways to ascertain their relevance to myocarditis. These pathways may elucidate the potential mechanisms through which Sivelestat exerts its therapeutic effects on myocarditis.

### 2.4 Molecular docking analysis

AutoDock Vina (version 1.1.2) was used for molecular docking (MD) analysis of Sivelestat and key targets to verify its interaction activity. The method for MD analysis is as follows: (1) Ligand Preparation: Sivelestat’s 3D structure (SDF format) was obtained from PubChem, energy-minimized in Chembio3D, then processed in AutoDock Tools 1.5.7 (adding hydrogens, calculating charges, assigning atom types) and saved as PDBQT format with defined rotatable bonds. (2) Protein Preparation: Target protein structures were downloaded from the PDB (prioritizing human proteins, high structural similarity to original ligand/active ingredient, and high resolution). Original ligands and water molecules were removed in PyMOL 3.2.0. Proteins were then processed in AutoDock Tools 1.5.7 (adding hydrogens, calculating charges, assigning atom types) and saved as PDBQT format. (3) Docking Setup: The docking grid box (40 Å x 40 Å x 40 Å, spacing 1.000 Å) was centered on the original ligand position; if unavailable, near key reported amino acid residues. Other parameters used default settings. (4) Docking & Analysis: Molecular docking was performed using AutoDock Vina 1.1.2. Interaction modes were analyzed with PyMOL and Ligplot. A comprehensive summary of the key interactions, including interacting residues, hydrogen bond distances, and types of hydrophobic contacts, is provided in Supplementary Table S2.

### 2.5 Experimental validation

#### 2.5.1 Animals

In this study, 30 male BALB/c mice strains were selected to establish EAM models, aged 6–8 weeks and weighing 20 ± 2 g. This strain is sensitive to Th2 immune response and can stably reproduce T cell-mediated myocarditis, which is highly related to the autoimmune pathological mechanism of human viral or immune myocarditis. The model was induced by injecting α-myosin heavy chain (α-MyHC) peptide, which is a classic model in this field (Hua et al., 2020; Zdebik et al., 2024). This model simulates the molecular mimicry and autoantigen exposure mechanisms underlying human myocarditis. α-MyHC is a specific antigen in myocardium. Autoimmune reaction against it has been found in human myocarditis patients. The sample size of n = 6 per group was determined based on reported studies using similar EAM models and power analysis to ensure adequate statistical power to detect significant differences in primary outcomes. The mice were housed in standard ventilated polycarbonate cages (5 mice/cage). The mice were maintained under controlled environmental conditions, including a constant temperature range of 22–25 °C, relative humidity of 40%–50%, and a 12-h light/dark cycle. They were provided with unrestricted access to water and food, alongside adequate ventilation. Prior to experimentation, the animals underwent a 1-week acclimatization period. All experimental protocols adhered to the ethical standards set by the Laboratory Animal Ethics Committee of Kangpu Cornerstone (Shaanxi) Health Life Technology (Approval No. APU20250217006) and were conducted in compliance with the ARRIVE guidelines (https://www.nc3rs.org.uk/arrive-guidelines) (Wang T. et al., 2024). All efforts were made to minimize animal suffering, including the use of anesthesia for all surgical and invasive procedures and the implementation of humane endpoints.

#### 2.5.2 Experimental protocol

After a 1-week acclimatization period, 30 male BALB/c mice were randomly assigned to five groups (n = 6 per group) as follows: a Sham group, an EAM (experimental autoimmune myocarditis) group, and three EAM groups treated with Sivelestat sodium at low (50 mg/kg), medium (100 mg/kg), or high (200 mg/kg) doses (designated as EAM + Siv-L, EAM + Siv-M, and EAM + Siv-H, respectively). The BALB/c strain was selected due to its susceptibility to Th2 immune responses and its ability to consistently develop T cell–mediated myocarditis, which closely mirrors the pathological mechanisms of human autoimmune myocarditis (Tong et al., 2025). The EAM model was established based on a previously described method with minor modifications. Briefly, on days 0 and 7, mice received subcutaneous injections of 250 μg of α-myosin heavy chain peptide (Ac-RSLKLMATLFSTYASADR-OH) emulsified 1:1 (wt/wt) in complete Freund’s adjuvant (CFA). Sham group mice were injected with a PBS-CFA emulsion instead (Hua et al., 2020). Sivelestat sodium (Huilun, Shanghai, China) was administered via intraperitoneal injection beginning 6 h after the initial immunization and continued once daily for 14 consecutive days. The clinical dose of Sivelestat for acute respiratory distress syndrome (ARDS) is 4.8 mg/kg/day. Based on body surface area conversion, the equivalent dose in mice is approximately 60 mg/kg/day (Reagan-Shaw et al., 2008). Considering the body weight of BALB/c mice (20 ± 2 g) and consistency with previous animal studies, the low dose was set at 50 mg/kg/day (Geng et al., 2024; Nie et al., 2022; Xie et al., 2025; Yoshida et al., 2022). The medium (100 mg/kg/day) and high (200 mg/kg/day) doses were selected to explore dose-dependent effects. The timing of the initial administration was determined because neutrophils serve as the first line of innate immunity and can form NETs within 5–6 h to participate in clearing harmful pathogens. However, excessive release of NETs can lead to immune-mediated tissue damage (Wang H. et al., 2024). Therefore, Sivelestat intervention was initiated 6 h after the first immunization. The duration of administration was determined by considering the pathophysiology of the acute-phase immune response in myocarditis and the clinical recommendation that Sivelestat treatment should not exceed 14 days according to its prescribing information.

#### 2.5.3 Echocardiography

Transthoracic echocardiography was performed using the Vevo 2100 (Visual Sonics, Inc) imaging system on the 21st day with a 40 MHz sensor. Anesthesia was performed using 2% isoflurane. Chest fur was removed with a depilatory cream. To ensure that the body temperature is stable at 35.5–36.5 °C, as mentioned earlier, the para-sternum long-axis M-mode is used to standardize the transducer localization in the ventricle for short-axis M-mode imaging. To evaluate cardiac function, three consecutive cycles were estimated and the average was taken.

#### 2.5.4 ELISA analyses

Peripheral blood was collected by enucleation and incubated at room temperature for 30 min. Next, serum was separated by centrifugation (400 × g for 30 min at room temperature) and stored at −70 °C. IL-1β (ml098416), IL-6 (ml063159), TNF-α (ml002095), and cTn (ml302661) levels were measured using the aforementioned commercial kits following manufacturer′s instructions and analyzed in a blinded fashion (Mlbio, China).

#### 2.5.5 Histological analysis

The heart tissue from mice was fixed, dehydrated, and embedded in paraffin by soaking in 4% paraformaldehyde. For each animal, three transverse sections from the left ventricle were obtained at different levels (apical, mid, and basal). From each section, at least 5 non-overlapping fields were captured at 200× magnification for analysis. The level of inflammation was measured by the percentage of inflammatory cell infiltration at 200× magnification (Li et al., 2022): level 0 indicates no infiltration or necrosis, level I is between 1% and 25% infiltration or necrosis per section, level II is between 26% and 50% infiltration or necrosis per section, level III is between 51% and 75% infiltration or necrosis per section, and level IV is between 76% and 100% infiltration or necrosis per section. Each sample was analyzed histopathologically by two researchers who worked independently and were blinded to the group information. Moreover, the area of inflammatory cells was evaluated using Image-Pro Plus software (version 6.0), which was expressed as the ratio of the area of inflammatory cells to the total area by examining at least four images.

#### 2.5.6 Immunohistochemistry analysis

EAM mice were treated with varying doses of Sivelestat. Based on preliminary echocardiographic and histopathological findings demonstrating dose-dependent efficacy, a dose of 200 mg/kg was determined to be optimal and used in all subsequent experiments.

Myocardial tissue sections (4 μm) were subjected to immunohistochemical staining for TNF-α, IL-1β, and IL-6. Briefly, after deparaffinization, rehydration, and antigen retrieval (citrate buffer, pH 6.0, 95 °C for 15 min), sections were blocked with 5% normal goat serum for 1 h at room temperature and incubated overnight at 4 °C with primary antibodies against TNF-α (Affinity, #AF7014, 1:200), IL-1β (Affinity, #AF5103, 1:200), and IL-6 (Affinity, #DF6087, 1:200). Following incubation with HRP-conjugated secondary antibody (1:200, 1 h at 37 °C), immunostaining was visualized with DAB substrate, and sections were counterstained with hematoxylin. Images were acquired from five randomly selected fields per section at 200× magnification using a light microscope (Olympus, Japan). The percentage of positively stained area was measured using ImageJ software (NIH, United States) to quantify cytokine expression levels.

#### 2.5.7 Immunofluorescence analysis

To identify NETs, mouse myocardial tissue samples were immunostained for citrullinated histone 3 (Abcam, #ab281584, 1:1000) and Neutrophil Elastase (Affinity, #AF0010, 1:100). The samples were fixed with 4% paraformaldehyde, washed with PBS, and permeabilized with 0.25% (v/v) Triton X-100 in PBS for 10 min. After washing three times with PBS, the samples were incubated with a 2% BSA solution in PBST (PBS with 0.1% Tween 20) for 30 min. Then, primary antibody solutions diluted in 2% BSA solution in PBST were added and incubated for 2 h at 37 °C. The samples were washed with PBS three times, incubated with a secondary antibody diluted in 2% BSA solution in PBST at a 1:500 ratio for 1 h in the dark, and then washed with PBS. Finally, the samples were counterstained with DAPI (1 μL/mL) and visualized under a microscope.

#### 2.5.8 Western blotting

Myocardial tissue samples from 3 randomly selected mice per group were used for Western blot analysis. Tissue homogenates were prepared in ice-cold RIPA buffer (Cell Signaling #9806) with protease and phosphatase inhibitors. Protein concentrations were determined by a BCA assay (Cell Signaling #7780) using BSA standards. After SDS-PAGE separation, proteins were transferred to PVDF membranes. Membranes were blocked with 5% BSA (for phosphorylated targets) or 5% non-fat milk (for non-phosphorylated targets) and then incubated overnight at 4 °C with primary antibodies: citH3 (Abcam, #ab281584, 1:1000), NE (Affinity, #AF0010, 1:500), PI3K (Affinity, #AF6242, 1:500), p-PI3K (Tyr458, Affinity, #AF3242, 1:500), Akt (Affinity, #AF6261, 1:500), p-Akt (Ser473, Affinity, #AF0016, 1:500), Bcl-2 (Servicebio, #GB154380-100, 1:500), and Caspase-3 (Servicebio, #GB155600-100, 1:500) Species-matched HRP-conjugated secondary antibodies (Zenbio #511203/#511103, 1:8,000) were applied for 1 h at room temperature. Proteins were detected using chemiluminescence (Thermo, #34096) and imaged. GAPDH (Servicebio, GB15004, 1:3,000) was probed as the loading control after membrane stripping. For phosphorylated proteins (p-PI3K and p-Akt), the signal intensities were normalized to their corresponding total protein levels (p-PI3K/PI3K and p-Akt/Akt) to accurately reflect pathway activation. For non-phosphorylated proteins (NE, Cit-H3, Bcl-2, Caspase-3), expression levels were normalized to GAPDH. Band intensities were quantified as background-subtracted integrated density values using ImageJ (1.8.0) by two independent investigators.

#### 2.5.9 Scanning electron microscopy (SEM) analysis

Mouse myocardial tissue was fixed with 4% (w/v) glutaraldehyde (dissolved in 0.1 M phosphate buffer, pH 7.4) for 2 h at room temperature and then cut into small pieces. The tissue was washed with buffer solution and subsequently fixed with 1% (w/v) osmium tetroxide (osmium acid) (dissolved in 0.1 M phosphate buffer, pH 7.4) for 2 h at room temperature. The samples were successively dehydrated using an ethanol gradient (50%, 70%, 90%, 100%), dried at the critical point, and coated with a thin gold film. The processed samples were examined using a scanning electron microscope (Thermo Fisher Scientific, Quattro S).

#### 2.5.10 TUNEL assay

Cell apoptosis was assessed *in vivo* using the TUNEL assay (Apoptosis Assay Kit, Vazyme Biotech Co., Ltd., Nanjing, China) following the manufacturer’s protocol. Tissue sections from all groups were fixed by immersion in 4% paraformaldehyde (pH 7.4) for 15 min at room temperature. Samples were then incubated with 100 μL of Proteinase K solution (20 μg/mL) for 20 min. Subsequently, 100 μL of 1 × Equilibration Buffer was applied for sample equilibration and incubated for 15 min. Following this, TdT incubation buffer was added, and slides were placed in a humidified chamber (wet box) and incubated at 37 °C for 60 min. Finally, nuclei were counterstained with DAPI (Beyotime Biotechnology, Shanghai, China) for 5 min. Fluorescence images were acquired using a microscope (BX53, Olympus, Japan).

#### 2.5.11 Quantitative real-time PCR (qPCR)

Total RNA was extracted from myocardial tissue using the RNeasy mini kit (Qiagen, Hilden, Germany). cDNA was synthesized using the QuantiTect Reverse Transcription Kit (Qiagen, Hilden, Germany). Quantitative PCR was performed using SYBR Green qPCR mix (Eurogentec, Seraing, Belgium) on a Roche LightCycler® 96 System (Roche Diagnostics, Basel, Switzerland). *Rplp0* (also known as 36B4) was selected as the reference gene based on its validated stability in mouse myocardial tissue (Ren et al., 2021; Takaoka et al., 2024). Relative gene expression was calculated using the ΔΔCt method. Primer sequences are listed in Table 1.

**TABLE 1:** Primer sequences for qPCR.

Gene	Forward primer (5'→3′)	Reverse primer (5'→3′)
*TNF*	CTG​AAC​TTC​GGG​GTG​ATC​GG	GGC​TTG​TCA​CTC​GAA​TTT​TGA​GA
*MMP9*	GGA​CCC​GAA​GCG​GAC​ATT​G	CGT​CGT​CGA​AAT​GGG​CAT​CT
*PTGS2*	CCA​CCT​CTG​CGA​TGC​TCT​TC	CAT​TCC​CCA​CGG​TTT​TGA​CAT​G
*IL-17*	ATC​CCT​CAA​AGC​TCA​GCG​TGT​C	GGG​TCT​TCA​TTG​CGG​TGG​AGA​G
*Rplp0*	TCT​CCA​GAG​GCA​CCA​TTG​AAA	CTCGCTGGCTCCCACCTT

## 3 Statistical analysis

All data are presented as mean ± standard error of the mean (SEM). Normality of data distribution was assessed using the Shapiro-Wilk test. For comparisons between two groups with normal distribution, Student's t-test was used. For data not following a normal distribution, the Mann-Whitney U test was applied. Comparisons between multiple groups were performed using one-way analysis of variance (ANOVA) with the Bonferroni post-hoc test for parametric data that passed normality and homogeneity of variance tests. For non-parametric data, the Kruskal-Wallis test with Dunn’s post-hoc test was used. Pairwise comparisons between groups were performed, with adjustment for multiple comparisons as appropriate. *P* values <0.05 were considered to indicate statistical significance. Statistical analyses and graphical representations were performed using GraphPad Prism 8.0 (GraphPad Software, San Diego, CA).

## 4 Results

### 4.1 Network pharmacology-based analysis

**FIGURE 1:**
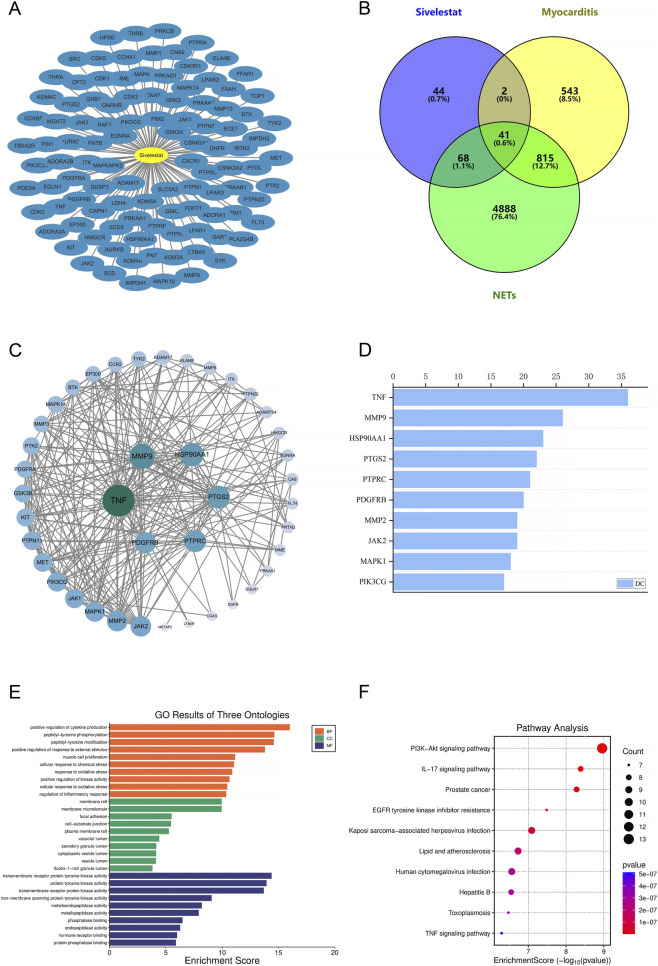
Analysis of the targets associated with Sivelestat, NETs, and myocarditis. **(A)** Network diagram of ‘Sivelestat-target’. **(B)** Venn diagram of Sivelestat, NETs and myocarditis-associated targets. Includes 155 Sivelestat-related targets, 1,401 myocarditis-related targets, 5,812 NETs-related target, and 41 Sivelestat-myocarditis-NETs-related targets (center). **(C)** Sivelestat anti-myocarditis PPI network. A larger area indicates larger nodes, a Green color indicates higher association, lighter color less association, and the core target is the target in the inner circle. **(D)** Degree values of core anti-myocarditis targets in Sivelestat; the X/Y axes respectively represent the Degree value and the target name. **(E)** GO enrichment analysis. Fold enrichment (y-axis), term (x-axis); green, orange, and purple represent the 10 core results for BP, CC, and MF, respectively. **(F)** KEGG pathway enrichment analysis (DAVID). Pathways (Y-axis), FDR (X-axis), and P-values (color change). Bubble size indicates the number of genes enriched in the pathway.

To explore the potential molecular targets of Sivelestat in the management of myocarditis, we performed a network pharmacology analysis (Figure 1). Initially, 155 genes associated with Sivelestat (SMILES: CC(C)(C)C(=O)OC1 = CC = C(C = C1)S(=O)(=O)NC2 = CC = CC = C2C(=O)NCC(=O)O) were identified using the SwissTargetPrediction database, applying a filter of “probability >0,” and the SEA database, with a “MaxTC” threshold greater than 0.3 as the screening criterion (Figure 1A). Concurrently, we retrieved 1,401 myocarditis-associated genes from the GeneCards and OMIM databases. Additionally, 5,812 NETs-related target genes were identified through searches of the GeneCards and OMIM databases. Subsequently, a Venn diagram was generated using the Microbiology Letter platform, resulting in the identification of 41 related targets (Figure 1B). The list of these 41 overlapping genes is provided in Supplementary Table S1. To visually assess the impact of Sivelestat on myocarditis, we constructed a PPI network consisting of 41 nodes and 243 edges, employing the identified intersecting genes (Figure 1C). The primary targets of Sivelestat involved in its anti-myocarditis effect are illustrated (Figure 1D), which comprises ten proteins. Notably, TNF exhibited the highest number of connections, being linked to 36 additional proteins. Based on their associations, *MMP9*, *HSP90AA1*, *PTGS2*, *PTPRC*, *PDGFRB*, *MMP2*, *JAK2*, *MAPK1*, and *PIK3CG* were linked to 26, 23, 22, 21, 20, 19, 19, 18, and 17 additional proteins, respectively.

To provide a comprehensive and systematic illustration of the potential mechanisms of action of Sivelestat in treating myocarditis, we conducted a GO enrichment analysis of its therapeutic targets at different levels using the DAVID database. The analysis covered BP, CC, and MF. We identified 340 statistically significant GO terms, comprising 223 BP, 38 CC, and 69 MF terms. Bar charts were used to display the top 10 enrichment terms of BP, CC, and MF with the highest gene counts (Figure 1E). Our results showed that the targets of Sivelestat in treating myocarditis were predominantly enriched in biological processes such as positive regulation of cytokine production, peptidyl-tyrosine phosphorylation, peptidyl-tyrosine modification, positive regulation of response to external stimulus, and other biological responses. They were also enriched in cellular components such as membrane raft, membrane microdomain, focal adhesion, cell-substrate junction, and other cellular components. Moreover, Sivelestat targets were found to exhibit molecular functions such as transmembrane receptor protein tyrosine kinase activity, protein tyrosine kinase activity, transmembrane receptor protein kinase activity, non-membrane spanning protein tyrosine kinase activity, and other molecular functions.

To systematically investigate the potential mechanisms underlying the anti-myocarditis effects of Sivelestat, we utilized KEGG pathways to predict the signaling pathways of Sivelestat’s potential anti-myocarditis targets. The results revealed 195 statistically significant related pathways. The top ten pathways with the highest number of associated genes are presented in Figure 1F. The top three potential pathways are the PI3K-Akt signaling pathway, the IL-17 signaling pathway, and the prostate cancer pathway. We focused on the PI3K-Akt signaling pathway based on the results of the enrichment analysis of core targets and pathways. These findings imply that Sivelestat can exert anti-myocarditis effects via a variety of mechanisms, among which the PI3K-Akt signaling pathway plays a key role in Sivelestat’s anti-myocarditis action.

### 4.2 Molecular docking and analysis

**FIGURE 2:**
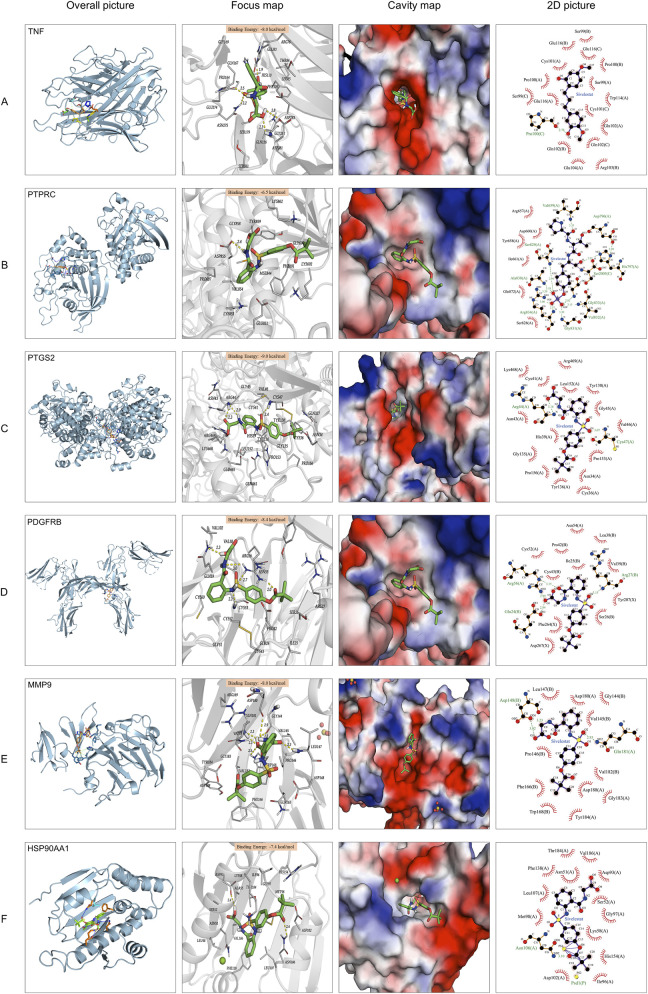
The picture of molecule docking model. **(A–F)** Molecular docking of Sivelestat with *TNF, PTPRC, PTGS2, PDGFRB, MMP9*, and *HSP90AA1*. The hydrogen bonds were represented by yellow dotted lines, and the length was marked around the lines.

Molecular docking was performed using AutoDock Vina (Trott and Olson, 2010). Docking was repeated three times, and the binding affinity of Sivelestat to six key targets was evaluated: *TNF, PTPRC, PTGS2, PDGFRB, MMP9*, and *HSP90AA1*. The ligand used for docking was Sivelestat. Among all the molecular docking results, the recorded maximum binding energy was −6.5 kcal/mol, and the minimum binding energy was −9.0 kcal/mol (Table 2). The binding energies of Sivelestat to the key targets were all less than −5.0 kcal/mol, indicating that these targets have good binding affinity and are potential candidate targets of Sivelestat. It is notable that the binding energy between Sivelestat and *PTGS2* is the lowest (−9.0 kcal/mol), indicating that *PTGS2* is a promising target for the anti-myocarditis effect of Sivelestat. The conformation with the lowest binding energy was visualized using PyMOL v2.2.0 (Figure 2). Notably, a detailed analysis of the binding interactions revealed that Sivelestat forms multiple strong hydrogen bonds and extensive hydrophobic contacts with key residues within the binding pocket of each target, with a comprehensive listing provided in Supplementary Table S2.

**TABLE 2:** Docking binding energy results of key targets and main active components (kcal/mol).

Ligand	Target	PDB ID	Affinity (kcal/mol)
Sivelestat	TNF	4zch	−7.7
	PTPRC	1ygu	−6.5
	PTGS2	5f19	−9.0
	PDGFRB	3mjg	−8.4
	MMP9	1itv	−8.0
	HSP90AA1	1byq	−7.4

### 4.3 Animal experiment

#### 4.3.1 Sivelestat improved cardiac function in EAM mice

**FIGURE 3:**
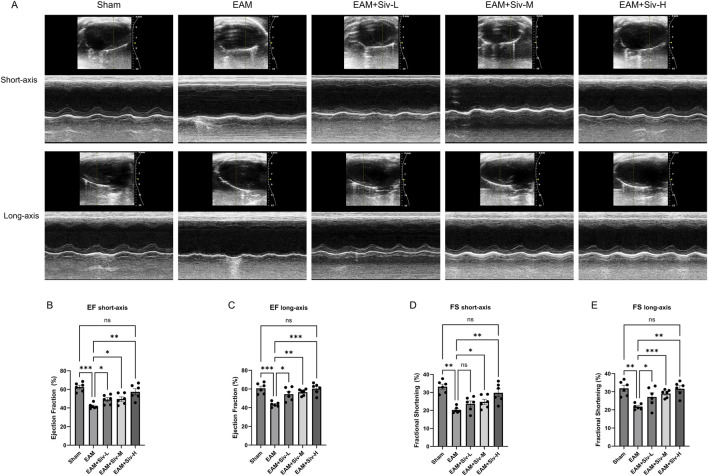
**(A)** Representative short-axis and long-axis M-mode echocardiographic images at day 21 post-immunization. **(B–E)** Quantification of left ventricular ejection fraction (LVEF) and fractional shortening (LVFS) from short-axis **(B,D)** and long-axis **(C,E)** views. Data are mean ± SEM (n = 6). (Sham vs. EAM and EAM vs. EAM + Siv). *p < 0.05, **p < 0.01, ***p < 0.001; ns, not significant (one-way ANOVA with Bonferroni’s post-hoc test).

In this study, we evaluated the cardiac function and other cardiac indicators of each group of mice through echocardiography. Cardiac function was assessed by long-axis and short-axis M-mode continuous echocardiography (Figure 3). In the EAM group, left ventricular ejection fraction (LVEF) and fractional shortening (FS) were significantly lower than those in the Sham group (*p* < 0.01), indicating that the myocarditis model successfully induced functional injury. Compared with the EAM group, both EF and FS of the mouse hearts improved after treatment with Sivelestat. However, there was no significant difference in FS in the short-axis EAM + Siv-L group (Figure 3D). The EAM + Siv-H group showed significant improvements in both EF and FS compared to the EAM group (*p < 0.01*). In addition, a dose effect was also observed: EF and FS showed an upward trend with the increase in the dose of Sivelestat (EAM + Siv-L < EAM + Siv-M < EAM + Siv-H).

#### 4.3.2 Sivelestat inhibits the formation of NETs in EAM mice

**FIGURE 4:**
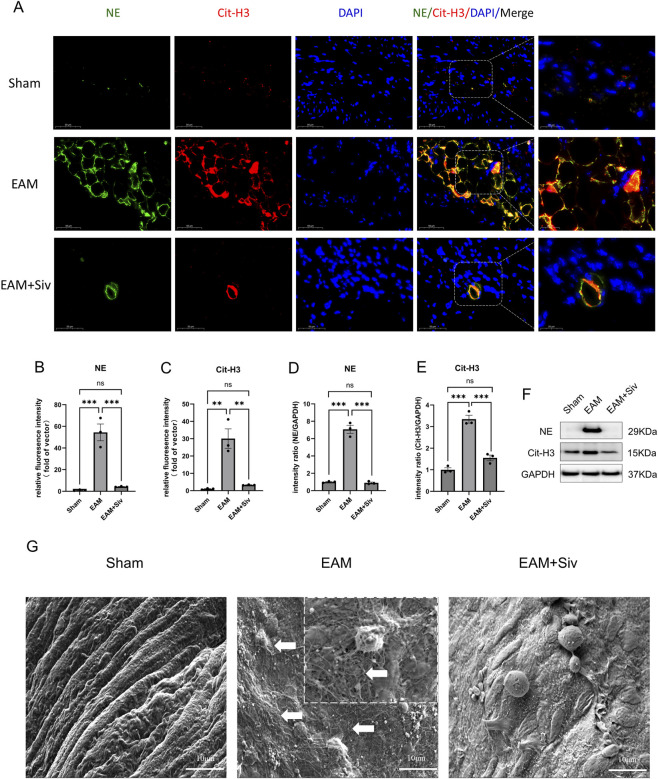
Sivelestat inhibits NETosis in EAM mice. **(A)** Representative immunofluorescence images of NETs (NE in green, Cit-H3 in red, DAPI in blue). Scale bar: 50 μm. **(B,C)** Quantification of NETs per area. **(D,E)** Western blot of NE and Cit-H3. **(F)** Densitometric analysis normalized to GAPDH. **(G)** Scanning electron microscopy (SEM) image showing NETs (white arrow). Data are mean ± SEM (n = 3 for Western blot). (Sham vs. EAM and EAM vs. EAM + Siv [200 mg/kg]) *p < 0.05, **p < 0.01, ***p < 0.001; ns, not significant (Kruskal-Wallis test for NETs counts; one-way ANOVA for Western blot).

NETs have been demonstrated to play a role in the pathogenesis and progression of myocarditis. In this study, we first applied immunofluorescence to detect NETs biomarkers (Cit-H3, NE) in myocardial tissue. As shown in Figures 4A–C, compared to the Sham group, significant NETs formation was observed in the myocardial tissue of the EAM group. This formation was markedly reduced following treatment with Sivelestat. Subsequently, we employed Western blot analysis to detect the protein expression of NETs biomarkers (Cit-H3, NE) in the mouse myocardial tissue, further validating the above findings. Figures 4D–F demonstrates that, compared to the Sham group, the EAM group exhibited substantial expression of Cit-H3 and NE proteins in the myocardial tissue. Treatment with Sivelestat significantly decreased the expression of both Cit-H3 and NE. Finally, we observed the mouse myocardial endocardium using scanning electron microscopy (SEM), yielding consistent results.

#### 4.3.3 Sivelestat alleviates the inflammatory response in EAM mice

**FIGURE 5:**
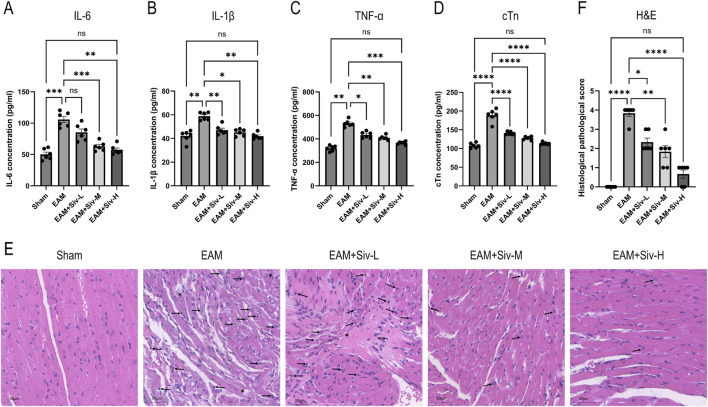
Sivelestat mitigates the inflammatory response and cardiac damage in EAM mice. **(A–D)** Serum levels of IL-6 **(A)**, IL-1β **(B)**, TNF-α **(C)**, and cTn **(D)** by ELISA. **(E)** Representative H&E-stained myocardial sections. Asterisks: necrosis; arrows: inflammatory infiltration. Scale bar: 50 μm. **(F)** Semiquantitative inflammatory score. Data are mean ± SEM (n = 6). (Sham vs. EAM and EAM vs. EAM + Siv). *p < 0.05, **p < 0.01, ***p < 0.001, ****p < 0.0001; ns, not significant (one-way ANOVA with Bonferroni’s post-hoc test).

IL-6, IL-1β, TNF-α, and cTn are key inflammatory factors closely associated with the pathogenesis and progression of myocarditis. To evaluate the systemic inflammatory response, we measured the serum levels of these factors in each group by ELISA. As shown in Figure 5, compared with the Sham group, the EAM group exhibited significantly elevated serum levels of IL-6 (Figure 5A), IL-1β (Figure 5B), TNF-α (Figure 5C), and cTn (Figure 5D). Intervention with sivelestat sodium markedly reduced the levels of these inflammatory factors compared to the EAM group. Furthermore, the reductions were dose-dependent; notably, the serum levels of IL-1β, IL-6, TNF-α, and cTn in the high-dose sivelestat group EAM + Siv-H were significantly lower than those in the EAM group and showed no statistically significant difference from the Sham group.

Next, we used H&E staining to assess myocardial injury, with representative images shown in Figure 5F. In the sham surgery group, myocardial cells were arranged neatly and orderly, with no evidence of edema or inflammatory cell infiltration in the interstitium. In contrast, the EAM group exhibited marked myocardial damage, characterized by edema, structural disarray, a substantial increase in inflammatory cell infiltration, and occasional focal fibrotic necrosis.

Treatment with sivelestat sodium ameliorated these pathological changes. Compared with the EAM group, the EAM + Siv-L, EAM + Siv-M, and EAM + Siv-H groups all showed reduced myocardial cell edema and inflammatory cell infiltration. This improvement was dose-dependent, as reflected by the pathological scores (Figure 5E). Notably, myocardial injury in the high-dose EAM + Siv-H group was significantly attenuated, with decreased inflammatory cell infiltration and a more orderly arrangement of myocardial cells.

**FIGURE 6:**
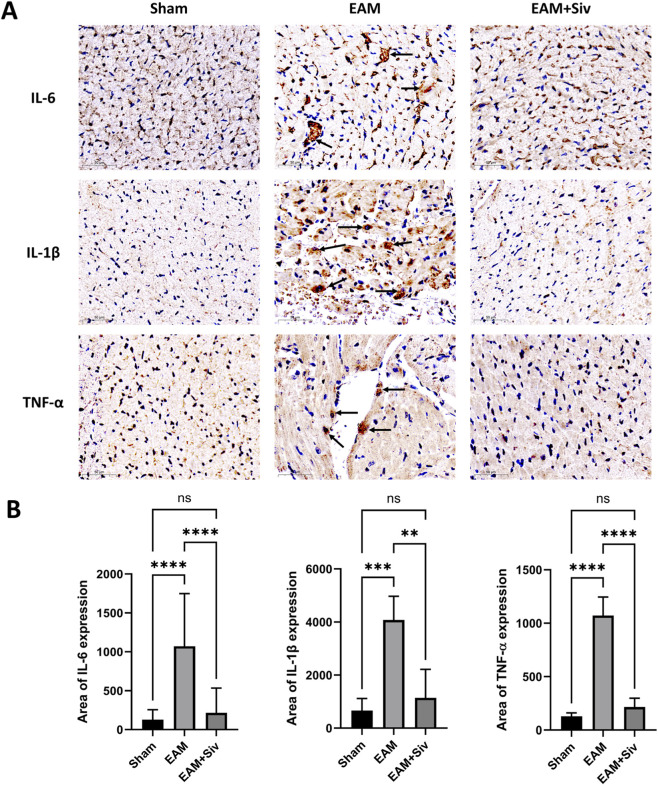
Sivelestat reduces the expression of IL-6, IL-1β, and TNF-α in the myocardial tissue of EAM mice. **(A)** Representative immunohistochemical staining. Brown: positive signal. Arrows indicate inflammatory infiltration. Scale bar: 50 μm. **(B)** Quantification of positive area percentage. Data are mean ± SEM (n = 3). (Sham vs. EAM and EAM vs. EAM + Siv [200 mg/kg]). *p < 0.05, **p < 0.01, ***p < 0.001, ****p < 0.0001; ns, not significant (one-way ANOVA with Bonferroni’s post-hoc test).

We then used immunohistochemistry to evaluate the myocardial infiltration of IL-6, IL-1β, and TNF-α in the Sham, EAM, and EAM + Siv (200 mg/kg) groups. The results (Figures 6A,B) demonstrated that, compared with the Sham group, the levels of these cytokines in cardiac tissue were significantly elevated in the EAM group. However, treatment with sivelestat (200 mg/kg) markedly reduced their expression. This tissue-level evidence is consistent with the serum cytokine profiles shown in Figures 5A–C and further supports our conclusion that sivelestat attenuates the local inflammatory response in the myocardium.

#### 4.3.4 Sivelestat alleviated the apoptosis by PI3K-Akt pathway activation

**FIGURE 7:**
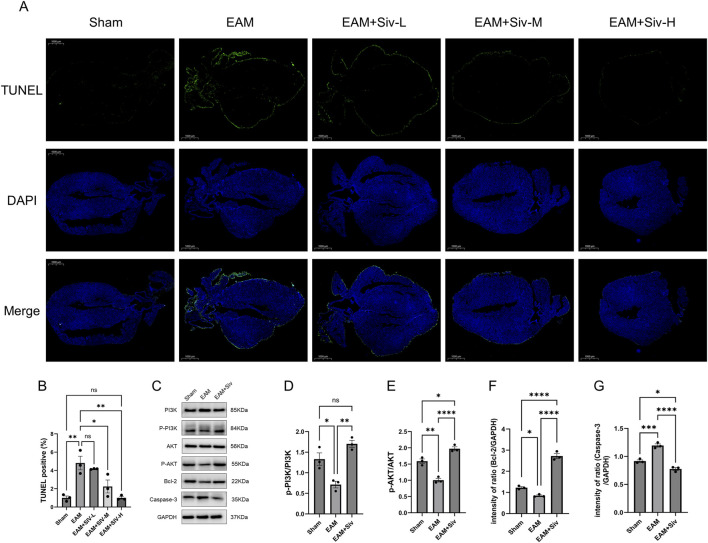
Sivelestat alleviates cardiomyocyte apoptosis and activates the PI3K-Akt signaling pathway in EAM mice. **(A)** TUNEL staining (green apoptotic nuclei, blue DAPI). Scale bar: 1,000 μm. **(B)** Quantification of TUNEL-positive cells/HPF in subepicardial region. **(C)** Representative Western blot bands. **(D–G)** Quantification of p-PI3K/PI3K, p-Akt/Akt, cleaved caspase-3, and Bcl-2. Data are mean ± SEM (n = 3 for Western blot). (Sham vs. EAM; TUNEL: EAM vs. EAM + Siv 50/100/200 mg/kg; Western blot: EAM vs. EAM + Siv 200 mg/kg). *p < 0.05, **p < 0.01, ***p < 0.001, ****p < 0.0001; ns, not significant (one-way ANOVA with Bonferroni’s post-hoc test).

TUNEL staining revealed that Sivelestat treatment significantly reduced the number of TUNEL-positive cells in EAM, counteracting α-MyHC peptide-induced apoptosis *in vivo* (Figures 7A,B). In the EAM group, TUNEL-positive apoptotic cardiomyocytes were predominantly localized in the peripheral (subepicardial) region of the myocardial tissue sections (Figure 7A). Sivelestat treatment significantly reduced the number of TUNEL-positive cells in this region compared to the EAM group. Western blot analysis demonstrated that Sivelestat reversed the apoptotic profile via the PI3K-Akt pathway: it increased phospho-Akt (p-Akt) and Bcl-2 expression while reducing caspase-3 levels, in contrast to the EAM group’s caspase-3 upregulation and Bcl-2 downregulation (Figures 7C–G). Collectively, these findings indicate that Sivelestat exerts anti-apoptotic effects in EAM *in vivo*.

#### 4.3.5 Sivelestat downregulates the mRNA expression of key network pharmacology-predicted targets

**FIGURE 8:**
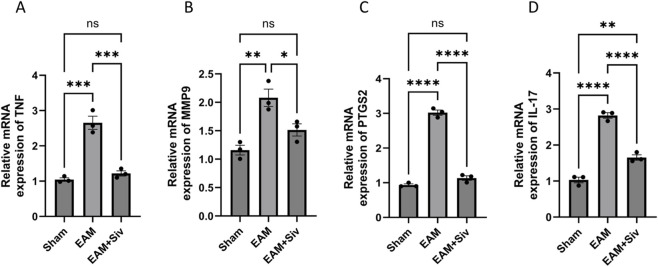
Sivelestat downregulates the mRNA expression of network pharmacology-predicted targets in the myocardium of EAM mice. **(A–D)** Relative mRNA expression of *TNF* **(A)**, *MMP9* **(B)**, *PTGS2* **(C)**, and *IL-17* **(D)** in myocardial tissues was determined by qPCR and normalized to Rplp0 (known as 36B4). Data are mean ± SEM (n = 3, each in triplicate). (Sham vs. EAM, EAM vs. EAM + Siv [200 mg/kg]). *p < 0.05, **p < 0.01, ***p < 0.001, ****p < 0.0001; ns, not significant (one-way ANOVA with Bonferroni’s post-hoc test).

To experimentally validate the network pharmacology predictions, we performed qPCR to assess the mRNA expression levels of key predicted targets (*TNF*, *MMP9*, *PTGS2*) and the core effector of the IL-17 signaling pathway (*IL-17*) in myocardial tissues. As shown in Figure 8, compared with the Sham group, the EAM group exhibited significantly upregulated mRNA expression of *TNF*, *MMP9*, *PTGS2*, and *IL-17* (*p* < 0.01). Treatment with Sivelestat (200 mg/kg) significantly reduced the expression of all four genes (*p* < 0.05 or *p* < 0.01). These findings provide transcriptional validation of the core targets identified by network pharmacology, consistent with the molecular docking prediction of high-affinity binding between Sivelestat and *PTGS2* (−9.0 kcal/mol), and suggest that Sivelestat exerts its cardioprotective effects through modulation of these targets.

## 5 Discussion

This study employed an integrated strategy combining network pharmacology, molecular docking, and *in vivo* validation to elucidate the therapeutic potential and underlying mechanisms of Sivelestat in myocarditis. Our findings indicate that Sivelestat exerts multi-target and multi-pathway effects, primarily by regulating NETs-associated pathways and the PI3K-Akt signaling pathway, significantly improving cardiac dysfunction and inflammatory responses in an EAM mouse model.

### 5.1 Suppression of NETosis as a primary cardioprotective mechanism

Our results confirmed that Sivelestat significantly reduces NETs formation (Figure 4) in mice with myocarditis by inhibiting NE activity, thereby improving cardiac function and diminishing myocardial inflammation. This finding aligns with previous studies demonstrating that targeted inhibition of NETs formation by interfering with structural components (NE, MPO, Cit-H3, PAD4, etc.) or dissolving NETs-DNA scaffolds with DNase I effectively reduces cardiac inflammation and improves cardiac function in myocarditis models (Li et al., 2022; Klopf et al., 2021; Kostin et al., 2024; Ichimura et al., 2024). Xi Zhang et al. (Zhang et al., 2025), demonstrated that inhibiting NETosis using DNase I or calcium channel blockers (e.g., verapamil) reduces cardiomyocyte apoptosis and improves cardiac function after acute myocardial infarction in mice, supporting our finding that NETs inhibition mitigates myocardial injury. Similarly, Carai, P. et al. (Carai et al., 2023), showed that in a Coxsackievirus B3 (CVB3)-induced viral myocarditis mouse model, neutrophils and NETs peaked in circulation and cardiac tissue at day 7 post-infection. Mice with specific PAD4 knockout, blocking NETs formation, exhibited significantly reduced cardiac damage, leukocyte recruitment, and inflammatory cell infiltration. Ge Mang et al. (Mang et al., 2024), further found that in a heart failure mouse model, the PAD4 inhibitor GSK484 suppressed NETs generation, improved cardiac function, and ameliorated cardiac fibrosis and inflammation. Building on these insights, we utilized an α-MyHC peptide-induced EAM mouse model. Immunofluorescence labeling (NE, Cit-H3), Western blot (NE, Cit-H3), and scanning electron microscopy revealed significantly increased NETs formation and substantial inflammatory cell infiltration in the myocardium of EAM mice compared to Sham controls. Sivelestat treatment markedly reduced myocardial NETs and alleviated inflammatory cell infiltration. These results strongly support our hypothesis that Sivelestat protects the myocardium by inhibiting NE activity and suppressing NETs formation, consequently reducing cardiomyocyte injury.

### 5.2 PI3K-Akt activation: bridging survival signaling and inflammation resolution

Our research further demonstrates that Sivelestat exerts anti-apoptotic effects by activating the PI3K-Akt signaling pathway, as evidenced by increased Akt phosphorylation (Figures 7D,E). This pathway regulates cell survival through downstream effectors, including the upregulation of the anti-apoptotic protein Bcl-2 and inhibition of Caspase-3 activation. Consistent with this mechanism, our data show that Sivelestat treatment significantly increased Bcl-2 expression (Figure 7F) and decreased Cleaved Caspase-3 levels (Figure 7G) in myocardial tissue. These findings confirm that Sivelestat inhibits cardiomyocyte apoptosis through PI3K-Akt pathway activation, thereby contributing to its cardioprotective effects in EAM mice, consistent with our network pharmacology enrichment analysis, which identified PI3K-Akt signaling as a core mechanism targeted by Sivelestat (Figure 1F). The role of PI3K-Akt as a critical regulator of cell survival and apoptosis is well-established across numerous cardiac pathophysiological contexts. For instance, Hongyu Geng et al. (Geng et al., 2024), reported that Sivelestat provided cardioprotection in septic myocardial dysfunction by activating the PI3K-Akt-mTOR axis, thereby reinforcing the plausibility of this mechanism in our model. Apoptosis, a programmed cell death process, is crucial for maintaining organismal homeostasis and regulating the cell cycle in multicellular organisms (Capela E Silva and Rodrigues, 2023). The PI3K-Akt signaling pathway is one of the most important intracellular transduction pathways regulating cell function and apoptosis; its activation or inhibition directly determines the suppression or promotion of apoptosis, as experimentally validated in numerous disease models. Studies indicate that the PI3K-Akt pathway plays a vital protective role in the heart, regulating cardiomyocyte survival, apoptosis, morphology, protein synthesis, and metabolic integration. For instance, in myocardial ischemia-reperfusion injury (MIRI) models, PI3K-Akt pathway activation synergistically enhances antioxidant, anti-inflammatory, and autophagic activity, thereby reducing cardiomyocyte apoptosis (Deng and Zhou, 2023). Notably, our study provides an additional layer of mechanistic insight by integrating this pathway with neutrophil-mediated inflammation. Beyond its direct anti-apoptotic role, PI3K-Akt activation has been implicated in modulating neutrophil function. Research in a diabetic microenvironment model demonstrated that activation of the ROS-PI3K-AKT-mTOR pathway could shift neutrophil activity from NETosis to phagocytosis, consequently reducing NETs formation and mitigating tissue damage (Guo et al., 2024). Given that our data confirm Sivelestat significantly suppresses NETosis (Figure 4), we propose a plausible, integrated mechanism: Sivelestat may alleviate myocardial injury not only by directly activating cardiomyocyte survival signals but also by indirectly attenuating neutrophilic inflammation via PI3K-Akt pathway modulation. This dual-action hypothesis, substantiated by our direct experimental evidence—including apoptosis reduction, Akt phosphorylation, and NETosis inhibition—provides a more comprehensive framework for understanding the drug’s therapeutic effect.

### 5.3 PTGS2 inhibition and IL-17 pathway modulation: experimental validation of network pharmacology predictions

**FIGURE 9:**
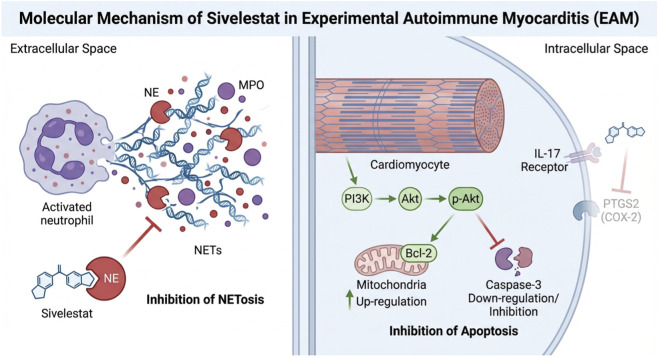
Schematic summary of the multi-layered mechanisms underlying Sivelestat’s cardioprotective effects in EAM mice. Sivelestat directly inhibits NE to block NETosis, serving as a core anti-inflammatory mechanism. It activates PI3K-Akt signaling to exert anti-apoptotic effects. Additionally, Sivelestat downregulates the mRNA expression of *PTGS2* and *IL-17*, suggesting potential involvement in broader immunomodulatory networks. Colored areas indicate mechanisms supported by direct experimental evidence; gray areas indicate mechanisms inferred from transcriptional evidence that require further functional validation. (Created with BioRender.com).

Network pharmacology analysis identified PTGS2 as a high-affinity binding target of Sivelestat (binding energy: −9.0 kcal/mol) (Table 2), and KEGG enrichment analysis highlighted the IL-17 signaling pathway as a top candidate (Figure 1F). In this study, we further validated these predictions using qPCR. Our results showed that myocardial *PTGS2* and *IL-17* mRNA levels were significantly elevated in EAM mice and were markedly reduced following Sivelestat treatment (Figure 8). *PTGS2*, which is highly expressed in macrophages, drives inflammatory injury by upregulating matrix metalloproteinases (including *MMP9*) and inhibiting GPX4 (An et al., 2025). Consistent with this, we observed that *MMP9* expression was also significantly suppressed by Sivelestat, suggesting potential coordinated regulation. In addition, the IL-17 signaling pathway is known to promote myocardial inflammation and fibrosis by recruiting inflammatory cells and activating cardiac fibroblasts (Huang, 2024). It is important to note that our validation at the mRNA level, while supportive, should be complemented by future studies confirming these changes at the protein level (e.g., by Western blot for PTGS2/COX-2 and ELISA for IL-17) to fully establish the translational impact of Sivelestat on these key mediators. Our data support the notion that Sivelestat may attenuate myocarditis through simultaneous modulation of PTGS2 and the IL-17 pathway. Furthermore, we validated TNF-α expression at the protein level (Figures 5C, 6), reinforcing the role of TNF as a core node in the network pharmacology analysis. Collectively, by integrating qPCR and protein-level data, this study provides multi-layered experimental support for the network pharmacology-predicted mechanisms of Sivelestat. The integrated mechanistic framework is summarized in Figure 9.

### 5.4 Integrated mechanistic summary

As summarized in Figure 9 and discussed in this section, our findings reveal a multi-layered mechanistic network underlying the cardioprotective effects of Sivelestat in experimental autoimmune myocarditis.

At the core mechanistic level, Sivelestat directly inhibits neutrophil elastase activity, effectively blocking the formation of neutrophil extracellular traps. This action fundamentally curtails the inflammatory cascade driven by neutrophil hyperactivation during the course of myocarditis, reducing both the direct injury to cardiomyocytes caused by NETs components and the amplification of local microenvironmental inflammation. This constitutes the cornerstone of Sivelestat’s cardioprotective effect.

At the synergistic level, Sivelestat activates the PI3K-Akt signaling pathway, exerting a dual protective function: (1) directly reducing cardiomyocyte apoptosis by upregulating the anti-apoptotic protein Bcl-2 and inhibiting Caspase-3 activation within cardiomyocytes, thereby preserving cardiac function; and (2) potentially modulating neutrophil function by shifting their phenotype from NETosis toward phagocytosis via the PI3K-Akt-related signaling axis within neutrophils, indirectly suppressing excessive NETs formation. This “pro-survival and anti-inflammatory” dual action reinforces the core mechanism, collectively promoting the resolution of myocardial inflammation and facilitating tissue repair.

At the potential regulatory level, network pharmacology analysis suggests that Sivelestat may exert additional protective effects by binding to *PTGS2* and modulating the IL-17 signaling pathway. *PTGS2*, as a key regulator of ferroptosis, represents another potential target; its inhibition could mitigate macrophage-mediated inflammatory injury, while downregulation of the IL-17 pathway might attenuate its activation of inflammatory cells and cardiac fibroblasts. Although the direct roles of these two pathways in the myocarditis model require experimental validation, existing studies suggesting potential crosstalk between PTGS2 and IL-17A signaling point to a possible supplementary mechanism for Sivelestat’s long-term cardiac protection, such as anti-fibrotic effects.

In conclusion, the protective effect of Sivelestat in the EAM model does not arise from the regulation of a single pathway but rather from an integrated, multi-target, multi-level protective network. This network is centered on the inhibition of NETosis, synergistically supported by PI3K-Akt activation, and potentially supplemented by the modulation of PTGS2/IL-17. This mechanistic framework not only deepens our understanding of Sivelestat’s pharmacological actions but also provides a novel theoretical basis for clinical therapeutic strategies for myocarditis. Future research employing cell-specific knockout models or combinatorial intervention experiments will be valuable to further validate the hierarchical contribution and temporal relationships among these pathways.

### 5.5 Limitations and future perspectives

Several limitations of this study should be acknowledged. First, while our integrated network pharmacology and molecular docking approach identified multiple potential targets including *TNF, PTPRC, PTGS2, PDGFRB, MMP9*, and *HSP90AA1* the experimental validation was primarily focused on protein-level analyses of targets most closely associated with the core mechanisms of NETosis and PI3K-Akt signaling. Although TNF-α was validated at the protein level in both serum and myocardial tissue, and *PTGS2* was supported by molecular docking and transcriptional evidence, direct mechanistic validation for other predicted targets (e.g., *PTPRC*, *PDGFRB*, *HSP90AA1*) is lacking. Specifically, the current study does not include cell-type-specific (e.g., macrophage, T cell, cardiac fibroblast) or pathway-specific (e.g., using siRNA, conditional knockout mice, or selective small-molecule inhibitors against these individual targets) experiments to establish definitive causality for each predicted target. This is a primary limitation. Second, the reliability of target predictions depends on literature and database mining, which may be influenced by data quality and update frequency. Third, although network pharmacology identified multiple myocarditis-associated pathways, the relative therapeutic contributions of these pathways remain unquantified. Despite these limitations, our study provides a robust multi-layered mechanistic framework for the cardioprotective effects of Sivelestat in autoimmune myocarditis. Future studies employing genetic approaches (e.g., tissue-specific knockout models) or pharmacological targeting of specific molecules will be necessary to dissect the precise contribution of each target to Sivelestat’s therapeutic effect in myocarditis.

## 6 Conclusion

In summary, our experimental results demonstrate that Sivelestat exerts cardioprotective effects during the inflammatory progression of EAM. Its mechanisms primarily involve inhibiting NETosis, activating the PI3K-Akt pathway to suppress apoptosis. Our computational analyses also suggest potential involvement of the PTGS2-IL-17A axis, which merits further investigation. Sivelestat is a multi-target candidate drug for the treatment of myocarditis. As an approved drug for ARDS, its safety database can accelerate the transformation of myocarditis indications.

## Data Availability

The raw data supporting the conclusions of this article will be made available by the authors, without undue reservation.

## Supplementary material

SUPPLEMENTARY TABLE S1 Information on key target genes.

SUPPLEMENTARY TABLE S2 Molecular docking interaction details.

SUPPLEMENTARY TABLE S3 Histopathological analysis: Scoring criteria and scores for each group.
